# Conference Report: THE SIXTH INTERNATIONAL MEETING ON CHEMICAL SENSORS Gaithersburg, MD July 22–25, 1996

**DOI:** 10.6028/jres.102.009

**Published:** 1997

**Authors:** Howard H. Weetall

**Affiliations:** Biotechnology Division, National Institute of Standards and Technology, Gaithersburg, MD 20899-0001

## 1. Introduction

The National Institute of Standards and Technology, an agency of the U.S. Department of Commerce, in cooperation with the 6th International Meeting on Chemical Sensors Steering Committee, held a symposium on chemical sensor science and technology on July 22–25, 1996 in Gaithersburg, Maryland. The meeting was attended by 427 persons from 27 countries. A total of 299 oral presentations and posters were given in three concurrent sessions over a period of 3 1/2 days. The sessions were divided into the following groupings: biosensors, gas sensors, humidity sensors, novel approaches to sensing, sensing principles and mechanisms, sensor fabrication technologies, acoustic sensors, electrochemical devices, optical devices, new materials, process control sensors, signal processing, and environmental monitoring. In addition there were four plenary lectures and two tutorial lectures.

Due to the size of this conference, not all sessions are summarized. An attempt has been made however, to give the reader a flavor of the nature of this meeting. The Proceedings will be published in the journal *Sensors and Actuators, Part B* within the next few months. If you are interested in obtaining more details, contact the journal directly or watch for the issue devoted to the meeting.

## 2. Plenary and Tutorial Lectures

The meeting began with an introductory lecture by Dr. Mary L. Good, Under Secretary for Technology at the Department of Commerce. Her lecture concerned the worldwide differences in protecting intellectual property rights and the role of the Department of Commerce in promoting American industry around the world. The theme of her presentation was that we should all “play on an even field.”

The first plenary lecture, given by David Walt of Tufts University was entitled, “Optical Array Sensor and Microsensors.” Walt discussed application of fiber optic bundles for producing arrays of different sensors on the same bundle using light sensitive polymerization techniques. He presented data on these arrays and discussed future applications using microfabrication and modification of the optical fiber bundles.

The second plenary lecture, given on the morning of the second day by Mark Meyerhoff of the University of Michigan was entitled, “Novel Non-Separation Enzyme-Immunoassay using Microporous Gold Electrodes.” This presentation dealt with the design and application of an electrochemical detection system that preferentially measures the surface-bound, enzyme-labeled antibody/ligand relative to enzyme-labeled reagent remaining in the bulk of the sample. The technique uses a nylon membrane between two chambers. The membrane is coated with gold on one side and as the second enzyme-labeled antibody passes through the membrane, it is picked up by the immobilized antigen bound to the first antibody. With substrate present in the same solution, the concentration of product at the surface of the gold membrane exceeds the level obtained in the bulk solution and is detected at the gold surface electrochemically as a response greater than the background signal.

The third plenary lecture, presented on the third morning by Ingemar Lundstrom of the Linkoping Institute of Technology, Linkoping, Sweden, was entitled, “Approaches and Mechanisms to Solid-State-Based Sensing.” This presentation dealt with the detection of hydrocarbon gases at high temperatures in auto exhausts and enumerated the difficulties with obtaining high efficiency. The system described, which has been tested in automotive gases, detects only 1 in 10^4^ molecules. Lundstrom discussed possible means of improving the efficiency of this system.

The final plenary lecture, presented on the last morning by Yoshiro Sakai of Ehime University, Ehime, Japan, was entitled, “Humidity Sensor Based on Polymer Thin Films.” Sakai discussed different types of polymer sensors, including resistive-type and capacitive-type humidity sensors. He discussed requirements for practical sensors with high selectivity, fast response time, little hysteresis, small temperature coefficient, long term stability and durability in organic vapors. He suggested solutions for each of these requirements.

There were two tutorial lectures. The first by D. Jed Harrison of the University of Alberta, Alberta, Canada was entitled, “MicroFabrication of Systems for Chemical Analysis: Integration of Fluid Flow, Chemical Reactions, Separation and Detection On-Chip.” Harrison discussed the requirements for, and difficulties with, the present state-of-the-art, and methods for producing chips capable of performing the work of a clinical chemistry laboratory. He also discussed the progress being made in his and other laboratories around the world.

The second tutorial lecture, presented by Michael Tarlov of the National Institute of Standards and Technology was entitled, “Chemical Sensing with Organic Monolayers.” The presentation included a discussion of the use of alkylthiols, silanes, and Langmuir-Blodgett films, and the use of patterning for the preparation of sensing systems containing organic materials, biological molecules and other types of sensing molecules.

## 3. Sessions Summaries

### 3.1 Gas Sensors, First and Second Sessions (reported by S. Semancik, U.S.A.)

Nine contributed papers were presented in these two technically-linked sessions by speakers who represented institutions in Germany, Great Britian, and the United States. The common theme of the presentations was the use of interesting and novel methods in gas sensing, and most presentations combined discussions of solid state detection techniques and fabrication technology. Two of the three presentations in the first session dealt with the advantageous use of varied temperatures in gas sensing. The first presentation described sensors that utilized a semiconducting metal oxide film as the active material, but created array formats by inhomogeneous heating of the oxide film, or by use of a graded-thickness SiO_2_ membrane that modified the gas access to sections of the metal oxide surface. Temperature differences of 50 °C were reported as sufficient to distinguish classes of organic compounds, and the prepared SiO_2_ membranes were indicated to be permeable enough to keep response times within the range of 10 s to 100 s. The next presentation described a temperature-modulated operational mode for an interdigitated capacitance structure with a heteropolysiloxane gas sensitive coating. The presentation focussed on CO_2_ detection. When combined with appropriate data processing, drift effects were eliminated and self-correction for humidity was provided. The last presentation of the first session dealt with enhancing selectivity by probing conductivity response at varied depths in semiconducting oxide films by using arrays of differently spaced electrodes within a single device. Differently sampled conduction paths gain information associated with the varied rates of combustion and diffusion of gases within the sensing material. The device described was self-diagnostic in the sense that degradation of the active material during operation could be monitored and the compensated for as changes occurred.

The second session contained six presentations representing a variety of detection principles and types of gas sensitive materials. The first two papers dealt with application of the field effect transistor (FET) as a gas sensor. The first paper concerned a capacitively controlled FET (CCFET) and the advantages that stem from separating the capacitor and the FET sections in the device. CMOS technology was used to fabricate the devices that included upper capacitor plates covered with Pd, Pt, SnO_2_, or Ga_2_O_3_ sensitive layers. Responses to H_2_ and NH_3_ at room temperature were described. The next paper concerned fabrication and operation of a PANIFET device, a field effect transistor with a polyaniline gate. The design employed a structure allowing simultaneous measurement of both work function and impedance changes. Methods of modifying the chemistry within the conducting polymer were discussed. This presentation also included the detection of H_2_, and NH_3_. Microfabrication of a solid-state device for measuring surface work function changes by the Kelvin probe technique was described in the third paper of the session.. This paper described the detection of O_2_, indicating that such a device holds the potential for a very rapid response. However, the prototype described was not appropriate for operation at atmospheric pressure.

The next two presentors discussed diode-based gas sensing schemes. The first discussed the fabrication and use of a thin film diamond diode structure with a catalyzed metal oxide electrode for O_2_ sensing. The use of diamond allows operation in harsh environments at temperatures considerably higher than those accessible to the other types of gas sensors. The other diode-based gas sensor for use in high temperature environments, described the use of Pd and SiC films. A portion of the presentation concerned the materials stability issues in operation, and the value of separating the Pd ans SiC layers with a SiO_2_ film.

The final presentation dealt with work on developing an ac impedance-based vapor sensor for organic solvents (including acetone, benzene, dichloromethane, tricholorethane, methanol, and hexane). The work described an interdigitated transducer with four electrodes fabricated and coated with phthalocyaninatonickel (II), cellulose derivatives, and other sensitive molecules. Responses to selected target vapors were discussed in relation to mechanistic aspects of gas-induced film modification and reversibility.

These two first gas sensor sessions were probably the most diverse. Sensing principles and detection enhancement methods included: temperature variation, varied spatial content electrical probing of films, conductivity measurements, FETs, work function change, diodes, and impedance monitoring (either separately or in combination). Due to the range of approaches, and in concert with the differing application targets, a wide variety of sensor construction materials (polymeric coatings, semi-conducting oxides, catalytic metals, and SiC and diamond. The content of these two sessions reflected not only the richness of important sensing problems, but also the richness of sensing research approaches being examined to find optimal solutions.

### 3.2 Gas Sensors, Third Session (reported by M. Post, Canada)

This session consisted of five papers representing a multinational forum of university and commercial collaborations from Germany, Romania, Spain, and Japan. The main common thread was the use of metal oxides as sensing materials. This is not innovative, but for several of the groups in particular, the focus was on the incremental improvement of these materials in their sensitivity and specificity to certain classes of gas analytes. The commercial potential for these improved materials was clear. The companies represented were were wide ranging and typical for these types of oxides. They included: H_2_, CO, NH_3_, SO_2_, NO_2_, and volatile organic compounds (VOCs). The non-specificity problem, that is known to exist for a large number of metal oxides with these gases, was addressed in a number a of different ways by the different groups. Research results with the oxides Ga_2_O_3_, MoO_3_, SnO_2_, and In_2_O_3_ were presented and claims of improvements out to ten fold in sensor response for selected gases, using sintered and/or thin-film overlayers of SiO_2_, (Pt, Pd), Rb_2_O, (Fe_2_O_3_, Ti, Au), respectively, were reported. The session was well attended with approximately 150 present for all presentations. Audience participation was high, and questions from the floor could easily have led to quite extended discussion periods beyond the time allotted.

### 3.3 Gas Sensors, Forth Session (reported by A. J. Ricco, U.S.A.)

The primary topics of this session were sensors suitable for the detection of exhaust gas species, such as CO, NO*_x_*, and hydrocarbons. Also included in this session were so-called “electronic nose” sensor systems. The presentations at the beginning of the session described the use of a pair of Y-stabilized ZrO_2_ sensors to detect both NO*_x_* and CO. There was some discussion of cross-sensitivity to propane, however, these sensors were found to have little sensitivity to H_2_O or CO_2_. Next a paper described several sensor array systems. The most intriguing, called MOSES or Modular Sensor System, was a rack-based unit designed to accept a variety of sensing modules based on various chemical sensor platforms, including quartz resonators, electrochemical sensors, SnO_2_-based devices and MOSFETs. Problems addressed using combinations of varied platforms included the discrimination of a variety of different coffees and plastic packaging materials. Critical to the success of this system for such pattern recognition tasks is an integral sampling system that delivers a precise volume of gas sample from the head space in each of a series of small, identical, sealed vials; the consequence is very repeatable concentrations of saturated head-space gas/vapor delivered to the sensor array, making the pattern recognition task easier than it might otherwise be for the neural network. One presentation described an array of two Au-doped gas sensors based upon WO_3_, SnO_2_, and ZnO semiconducting layers, each deposited upon a micromachined Si_3_N_4_ membrane with integral heater and temperature monitoring capabilities. Various gases can be detected using this array of conductivity-based sensors with appropriate pattern recognition. Species such as colognes, perfumes, and whiskey were differentiated from one another. Another paper described a system claimed to mimic mammalian olfactory responses using an array of conducting polymer-based sensors (polypyrrole, polythiophene, polyaniline etc) with various dopants. These electropolymerized film-containing array systems are available commercially from a few small British companies. The mechanism of operation of the sensor array is the change in conductivity of the polymer films that occurs when the absorption of molecules from the gas phase perturbs the local physical and electronic structure of the polymer chains. This is coupled with pattern recognition algorithms to identify different odors.

In summary, this session described the present state-of-the-art for sensor array technology presently used for detection and quantitation of gases/vapors containing multiple components by using several sensors with different specificities and sensitivities to produce a pattern unique to a specific complex gas mixture.

### 3.4 Gas Sensors, Fifth Session (reported by M. Egashira, Japan)

Four presentations in this session described semiconductor gas sensors based on metal oxides. The first described the utilization of scanning tunneling microscopy to characterize SnO_2_ gas sensing materials. The size of the SnO_2_ crystallines observed by scanning tunneling microscopy (STM) agreed well with x-ray diffraction (XRD) and tunneling electron microscopy (TEM) observations. However, rather round shaped STM images were observed due to the comparable size of the STM tips and the SnO_2_ crystallines. An interesting finding was that the stability of the STM images of pure and Ba-doped SnO_2_ was dependent upon the gas atmosphere between air versus nitrogen (though the details should be clarified in the future). From combined measurements of infrared (IR), x-ray photoelectron spectroscopy (XPS), capillary electrophoresis mass spectrometry (CEMS), and conductance changes of Cd^+1^ + XS^−^ and Sn*_x_*WO*_y_*-based sensors, it was deduced that the surface of metallic species act both as the centers determining the dark conductivity behavior, and as the sites for chemisorption of O_2_, SO_2_, and CO. The relative location of energy levels of the chemisorbed complexes in the and gap to the Fermi energy was considered as a determining factor for the observed different chemisorption mechanisms. Energy transfer to the sites with different coordination at the surface was also discussed in terms of a model based on a correlation between collective electronic characteristics of the crystal and local quantum mechanical parameters of the chemisorbed complexes. In another presentation, it was reported that surface exchange at semiconducting Ga_2_O_3_ thin film sensors were responsible for improving sensitivity and selectivity of the sensor. This paper discussed the semiconductivity for Ga_2_O_3_, that is stable even at high temperatures, and the changing of the chemical reactivity of the surface by sputter depositing a continuous layer of another metal oxide. Films of 30 nm to 300 nm thickness of the following metal oxides were prepared: WO_3_, AlVO_3_, V_2_O_3_, Ta_2_O_3_, SrTiO_3_, NiO, and TiO_2_ on a 2 μm thick Ga_2_O_3_ surface. With transition metal oxides, fundamental changes in gas sensitivity were observed due to their catalytic activity. Another important aspect was the change in electrical conduction that occurred via the Ga_2_O_3_ layer, or via the additional oxide layer. In still another presentation heteroepitaxy of SnO_2_ and ZnO were reported on silica on using oxide buffer layers. This kind of technology is very important in view of eventual integration of arrays of semiconductive gas sensing films on a silicon platform. In addition, the epitaxial films provide improved stability and novel sensing characteristics. To realize the epitaxial growth, and to ensure the electrical insulation between the silicon and the sensing oxide films, an intervening oxide buffer layer, such as CeO_2_ or ZrO_2_ was used and it was found that preferred orientations can be established.

### 3.5 Sensing Principles and Mechanisms, Session 1 (reported by A. D’Amico)

The session concerned the design and application of chemical analytical instruments and opened with a presentation on an instrument for analysis of Mars surface chemistry. The sensors proposed for this study are based on reflectivity changes of metal surfaces in the presence of chemical species that are expected in the Martian atmosphere. This paper was followed by a report on the influence of the dynamic responses of individual sensors on the overall performance of a sensor array. Fundamental aspects of the deconvolution process in a complex system of sensors including: quartz microbalance resonators and tin oxide sensors were considered. In another presentation, novel sensing principles were applied to electrochemical enzyme sensing. In bioelectrocatalysis the phenomenon does not consider redox mediators. The sensing principle was clearly illustrated and numerous molecular transducers for the development of biosensors were discussed. Examples of immunosensors based on bioelectrocatalysis using laccase and peroxidase transducers were mentioned. The next presentation dealt with high temperature oxygen sensors based on optical transmission properties of SrFeO_2.5+_*_x_* thin films, obtained by laser deposition of sintered material. At about 400 °C this material changes it transmittance by three orders of magnitude. Data was presented for both temperature and oxygen partial pressure. Reversibility and fast response times are the major features of this sensor. This presentation was followed by another dealing with optical sensing, using membranes. The light intensity is reflected by the sensitive membrane attached to the end of an optical fiber. The membrane is a swellable polymer. Different polymer types were discussed and their chemical specificities described. The last paper dealt with the gas sensing properties of CoO doped SiO_2_ nanocomposites. This material shows optic transmittance changes in the near infrared region in the presence of NO. A variety of sample structures were described and tested. The paper proposed some explanation of the sensitivity that appears to be related to the role played by the cobalt oxide.

### 3.6 Sensing Principles and Mechanisms, Session 2 (reported by W. Gopel, Germany)

This session dealt with the effects of metal atom/oxide interfaces and their influence on gas adsorption. The three phase boundary metal/oxide/gas leads to Schottky barrier formation, ohmic contacts or transfer reactions involving ion conduction. The first presentation focused on analysis of Pd/SnO_2_ interface effects that were discussed on a very broad basis and indicated the huge variety of possible experimental situations. The second presentation was far more specific; a careful analysis of Schottky barrier formation and its phenomenological description and evaluation of data was given. The link between interface characteristics and sensor phenomena was outlined in a later presentation with a variety of different case studies. The last presentation in the session illustrated the well-known sensitivity of work functions and conductivities to small variations in metal/oxide pretreatment for a given material combination. First guidelines to produce more stable interface electronic structures were presented, although the number of parameters to be adjusted was seen to be large in the choice of case studies discussed at this session.

### 3.7 Acoustic Sensors Sessions (report by D. L. Venezky, U.S.A.)

These sessions turned away from the usual surface acoustic wave (SAW) sensors with organic coatings to the use of solid metal oxides (SMO). The trio of papers in the first session reviewed the theoretical and experimental details of the application of SMO as an active coating for the detection of gases that in each paper was limited to H_2_S. The SMO under discussion was a thin film of WO_3_ on either a quartz or LiNbO_3_ dual delay line oscillator configuration. One report considered that the difference frequency and measurement of the film’s conductivity can be measured, and the sensitivity of the SAW chemiresistive gas sensor can be realized, if the film is treated as an ac impedance element. A comparison between a 40 MHz LiNbO_3_ and a 250 MHz quartz device operated at 250 °C indicated that the quartz was the preferred device material. Details of the temperature compensation of quartz and non-temperature compensation of LiNbO_3_ over a wide range of temperatures from –35 °C to about 565 °C was presented. For the best results, it was reported that 27° rotated Y-cut quartz had the highest temperature stability. One paper described the details of determining the best substrate temperatures for WO_3_ film deposition (from W sputtering in O_2_) and annealing, to obtain the best sensitivity and reproducibility.

The second session was devoted primarily to the quartz crystal microbalance (QCM); the parameters effecting its operation and applications. One report discussed studies at 15 MHz using QCMs with 1.1 mm thin films of rubbery and glassy materials, and reported the determination of the shear parameters of the thin layers. An important conclusion was that a QCM operating in a liquid media is more involved than one in a gaseous environment, and requires information about the liquid properties. It was stated during this session that the frequency changes of a coated QCM immersed in a liquid cannot differentiate between the mass changes in the film, the viscosity of the liquid, the density of the liquid or the viscoelastic properties of the film. The presentation described a QCM device having two electrode surfaces that were etched to give one rough and one smooth surface. The changing viscoelastic properties of the thin film on such a device minimally effected the observed frequency. Applications ofCM were illustrated by the determination of viscoelastic properties of polyacrylic acid in phosphate buffered solutions by observing frequency changes of impedance minima; by detection of organic solvents (toluene, trichloromethane, tetrachloroethane, tetrachloroethane, etc.) with a quartz crystal resonator (QCR) coated with organic polymers including a cross-linked polymer and, by detection (mass fraction <5×10^6^) of non-ionic (Triton X100) in “real” waste water in the presence of other organic polymers including: diethylene glycol, vinyl chloride, and organic peroxides. The last presentation of the session described a proposed multisensor for waste water analysis that would measure conductivity, pH, turbidity, temperature, O_2_, and Cl with a 10 MHz QCM.

### 3.8 Electrochemical Devices Sessions, First and Third Sessions (reported by B. Ramos, U.S.A.)

The first session began with a presentation on solid state amperometric sensors for NO*_x_*. The first report was on the use of NASICON (sodium ion conductor)/nitrite couple for amperometric sensing of NO_2_. When employing NASICON with NaNO_2_, an almost linear relationship for NO_2_ was obtained in the concentration range of 0 g/L to 80×10^–6^ g/L. Differences in sensing behavior were observed when using NASICON with NaNO_2_ and NaNO_3_, that were attributed to the differences in ion conductivity. This presentation was followed by a description of a high-temperature hydrogen sensor based on stabilized zirconia and a metal oxide electrode. An interesting sensor configuration was described that employed a half-open yttria stabilized zirconia tube. Pt paste was used as a reference electrode, and a ZnO sensing layer was formed on the outer surface of the tube that was covered with a Pt mesh. The device was sensitive to H_2_ with a 90 % response time as short as 5 s. The last presentation in the session discussed the development of improved CO sensors. In this paper the author described a stable Nafion™-based carbon monoxide sensor. The purpose of his study was to develop electrochemical CO sensors that are smaller and cheaper than the existing commercially available products. The focus of the investigation was on the possibility of using a planar design, mass production compatible fabrication, and replacement of sulfuric acid with a thin Nafion™ solid polymer electrolyte. The resulting sensor showed fast response to 90 % of steady state <20 s, linearity from 0 mol/L to 500×10^–6^ mol/L of CO, and low detection limit of <1×10^–6^ mol/L. A microfabricated electrochemical CO sensor was discussed. The sensor was made from photolithographically defined platinum electrodes deposited on alumina. A layer of platinum black was added, followed by liquid Nafion™ The response was found to vary with changes in humidity, so the sensor was designed to compensate for this dependence.

The third session included a discussion of a chemical microsensor for the detection of mercury ion based on chalcogenide glass thin films. The chalcogenide glass layer based on the system AgBr-Ab_2_S-As_2_S_3_ was described. These films were studied with x-ray diffraction, Auger spectroscopy, and tracer diffusion measurements. Potentiometric measurements were performed, and a detection limit close to 10^–8^ mol/L for mercury was obtained. A very good selectivity against Pb^+2^ and Cd^+2^ was demonstrated. The buffer-dependent response kinetics of a conductimetric pH sensor were elaborated on another presentation that involved a hydrogel copolymer of 2-hydroxyethyl methacrylate and N, N-dimethylaminoethylmethacrylate. The response kinetics agreed with established buffer-mediated diffusion reaction theory. In the final presentation, a solid state gas sensor for CO_2_ partial pressures was described that used potentiometry. There was discussion of how the ionic surface conductivity changes could be detected by application of a microelectrode impedance technique.

### 3.9 Electrochemical Devices, Second Session (reported by W. Heineman, U.S.A.)

This session included presentations on a thin film zirconia air-to-fuel ratio sensor capable of detecting reducing gases in oxidative and in reductive atmospheres. In another presentation a set of prototype electrochemical monitors for on-line measurement of O_2_, CO_2_, CO, NO, NO_2_, and methanol, applicable for medical, industrial and environmental monitoring was described. The common characteristic of these devices is the solid polymer electrolyte, in membrane form, with integral catalytic sensing, reference, and counter electrodes. A small hand held monitor was originally developed for a medical application to non-invasively monitor patients undergoing alcohol abuse treatment. A modification of this device was also used for measurement of methanol, which is an important gasoline fuel additive and also an important fuel in reformed methanol-air fuel cells. A battery powered monitor was also described. Another presentation described a carbon interdigitated array electrode consisting of 125 pairs of microbands having 2.5 mm width separated by 1.5 mm divisions. They were prepared by sputtering carbon films on to a Si/Si_3_N_4_ substrate. The behavior of these devices were comparable to that of glassy carbon. In those electrochemical applications discussed, a correlation of 0.999 was found between the thin-film carbon and the currently used systems. The next presentation discussed the properties of different surfaces of microelectrode arrays for heavy metal detection. On one sensor chip, four arrays and a counter/reference electrode are integrated. This device was capable of detecting Zn, Cd, Pb, and Cu with detection limits of 1 × 10^−10^ mol/L after a 300 s preconcentration step. In another presentation calix 4-arenediquine bound to alkylammonium cation produces a set of redox peaks at positively shifted potentials. The magnitude of the potential shift can be explained on the basis of stabilization due to the formation of hydrogen bonding in addition to the change of binding characteristics of ion-dipole to ion-ion interaction by electron transfer reaction. The magnitude of the hydrogen bonding correlates with the decrease of the potential enhancement from which the selective recognition of individual alkylamines can be made.

### 3.10 Novel Approaches to Sensing Sessions (reported by M. Butler, U.S.A.)

Oral presentations were made covering a wide range of topics. One paper described the mimicking of silkworm behavior in searching for another silkworm. This involved the use of a binocular pair of chemical sensors integrated with a fan to flow the air over the sensors and a shield to prevent cross-talk. This device was capable of accurately determining the direction of odor sources. It could be useful in searching for gas leaks and bad odor sources in buildings. Another presentation described a five sensor array of various metalloporphryrin layers on a quartz crystal microbalance to evaluate the freshness of a variety of foods. Several pattern recognition techniques were used to evaluate the data, and it was found that food quality could be easily distinguished. This was followed by a presentation describing a sensor array using surface photovoltage to evaluate the various components in the taste of wine. A variety of lipid membranes were used along with pattern recognition algorithms, and principle component analysis. Using nine different lipid membranes, five different white and rose wines could be unambiguously identified. Also described was a field effect transistor (FET) for detection of important, very toxic semiconductor processing gases, including PH_3_ and AsH_3_. The large dipole moment of these molecules makes this a particularly sensitive method. Additional papers reported on the combination of optical and electrochemical sensing methods to enhance sensor performance. These techniques included surface plasmon resonance combined with electrochemical stripping for the detection of trace metals in the environment. In another case, evanescent wave technology was combined with electrochemistry to develop a sensor for redox reaction detection. Microfabrication to produce simple arrays using both optical and electrochemical sensors was discussed. Screen printing to produce electrodes and semiconductors was described in several presentations. One paper described an electrode sensitive to ethanol vapor that when affected by a large voltage increase (causing heating) enhances the sensor signal several fold. This positive effect was used in a positive feedback that resulted in the development of a simple yet highly sensitive device that does not require a separate heating element.

The key running through most of the presentations was the need for and the development of arrays that utilize more then one technology such as optical and electrochemical approaches on the same device.

### 3.11 Biosensors, First Session (reported by Francis Ligler, U.S.A.)

The most intriguing of the presentations in this session concerned a DNA sensor based on absorptive stripping potentiometry. Screen printed electrodes were used to accumulate ng amounts of DNA or RNA in less than 2 min. The amount bound was quantitated during a constant-current stripping step in which the oxidation of bound guanine residues was measured. Modification of the electrodes with single-stranded DNA led to sequence-selective sensing. The electrodes are in the process of being coupled with hand-held analyzers for on-site monitoring.

### 3.12 Biosensors, Second and Third Sessions (reported by M. Aizawa, Japan)

Eight presentations were given in these two sessions. The first paper of the session described a glucose sensor based on metallocene-containing carbon paste. The authors described a nichocene electron mediator coupled with glucose oxidase for the analysis of serum glucose. Another paper reported on the direct amperometric detection of glucose using direct electron transferring through a charge transfer complex that the authors claimed was not a mediator. A report described human trials on a subcutaneously implanted, needle-type glucose sensor based on a Nafion™ outer layer coating. The sensor was fabricated by depositing diaminobenzene and glucose oxidase on the electrode surface, coating with Nafion and curing at room temperature. Performance of the sensor was discussed. A fourth glucose sensor was described using a thermally stable glucose oxidase extracted from *S. thermophilous*. This electrode showed long term stability.

The next biosensor session included two papers on ion-specific field effect transistor (ISFET) based biosensors with three papers on bioaffinity sensors. The fist enzyme sensor paper described a “rechargeable” glucose sensor, in which the enzyme could be replaced. The next enzyme paper described a disposable sensor for detection of esophageal acid using silk-screen printing as the method of fabrication. The ISFET-based biosensing systems described for coulometric sensing device for lysozyme determination. Another ISFET was described for the measurement of penicillin during fermentation using the enzyme penicillinase.

The last papers dealt with a fluorescence immunoassay system, in which the antibody is immobilized. The first of this group used antibodies immobilized in capillary tubes, another used antibody immobilized by avid-biotin to a support, and the last paper described antibody immobilized on polyvinylalchol-glutaraldehyde coated discs. The readout methods described were all different but none of them were innovative or new.

### 3.13 Biosensors, Fourth Session (reported by S. Glazier, U.S.A.)

This biosensor session included presentations that were applied in nature, and in some cases dealt with clinical situations. The first paper was directed at a biosensor for the detection of stroke. The detection of the D-dimer of fibrinogen is an indication of thrombus formation since it is cleaved in the formation of fibrin from fibrinogen. The paper discussed the development of fiber optic sensors that can be used in catheters for detection of this protein fragment. Another report described the development of a rapid surface photo-voltage image sensor and its application to the monitoring of biochemical reactions. This system utilizes digital data processing rather than analog processing found in the conventional surface photo-voltage sensor. The application of surface plasmon resonance (SPR) for HIV detection was presented. In this paper specific antigens to the HIV were detected and epitome maps prepared of two specific HIV antigens. The method is no more sensitive that radioimmunoassay, enzyme immunoassay or chemiluminescence assay. It is however, more expensive. There was one presentation on a non-dispersive infrared nitrous oxide detector capable of detecting the compound in the 10^–6^ mol/L range. The instrument was effective in concentrations of CO_2_ as high as 1500×10^–6^ mol/L and H_2_O as high as 70 % humidity content. The detector utilizes three channels, one for CO_2_, one for H_2_O, and the third channel is for nitrous oxide. The background can be eliminated from the signal. One paper described the use of immobilized thermolysin as a recognition element for Zn^+2^. The thermolysin was first immobilized on porous glass particles. A flow injection system contained a column with the immobilized enzyme. Product was detected spectrometrically. There was one interesting presentation on the use of electroluminescence for immunoassay. The assay is homogeneous using luminol-labeled antibodies that are inactivated at an electrode surface when complexed to the antigen.

### 3.14 Humidity Sensors, First and Second Sessions (reported by P. Huang, U.S.A.)

The presentations in these sessions on humidity sensors consisted of 15 oral reports and seven posters, representing the state-of-the-art in humidity measurement technology and standards. Topics covered new materials for sensors, new sensing techniques, new fabrication methods, and current standards for calibrating sensors.

The International Meeting On Chemical Sensors ’96 included for the first time, a session on trace moisture sensing and measurement in semiconductor gases. Trace moisture contamination in semiconductor processing gases directly affects quality and yield of semiconductor products like very large integrated circuit (VLIC) and its components. State-of-the-art sensing techniques including optical frost-point detection, acoustic resonator, and technology for sensing below the level of 10×10^–6^ mol/L of water in air based on permeation methods were also discussed in the sessions.

In general, the work presented both orally and in the posters, indicated that advanced materials and sensing techniques make it possible to detect humidity over a large range of interest with sufficient sensitivity. However, problems in stability and reproducibility still remain. Active research and development are continuing in this area around the world.

### 3.15 Humidity Sensors, Third Session (reported by D. Galipeau, U.S.A.)

The last session on humidity sensors covered a wide range of topics from humidity generators, to new materials and techniques to measure humidity. In the first presentation, a new flowmeter calibration method for use in a divided flow humidity generator was discussed. This method used a third flowmeter to measure total flow and statistical calculations to correct for flowmeter error. In the second presentation, optimization of Na-modified carbon films for use as humidity sensors was discussed. The sodium content and carbonization temperature and duration were found to significantly affect the humidity response. The third presentation described the use of polyphosphizine membranes as very sensitive humidity sensors. It was demonstrated that these materials had high sensitivities to both high and low humidities, with less than 5 % drift over 5 months. The fourth paper discussed a humidity sensitive field effect transistor fabricated using conventional silicon microtechnology. The threshold voltages of the device were found to decrease from 1.94 V to 1.52 V as the relative humidity was increased from 30 % to 90 %. An optical humidity detection technique using a metal oxide film was presented in the fifth talk. Thin films of Co_3_O_4_, Mn_3_O_4_, Fe_2_O_3_, NiO, and CuO were shown to have humidity sensitive optical absorption in the visible wavelength region at room temperature. The absorbance change was reversible with a response time of a few minutes. In the final paper, it was demonstrated that dew point measurement techniques based on a surface-acoustic-wave (SAW) sensor had significant performance advantages compared to commonly used optical reflection techniques. The advantages included higher measurement stability during the frost point transition, lower sensitivity to contamination, and improved resolution.

### 3.16 Sensor Fabrication Technology Sessions (reported by P. Hesketh. U.S.A. and N. Sheppard, U.S.A.)

In an invited presentation, the development of an automated hand held fiber optic biosensor consisting of a portable, four channel sensor system based on the evanescent wave sensing principle was described. The target analyte competes with a fluorescently labeled analog for binding sites immobilized to the fiber surface. The presentation concluded with a video of a field trial in which an air sampler, sensor, and control electronics were flown on a small remotely controlled, drone vehicle. The next presentation on the fabrication and packaging of integrated chemo-optical sensors using microfabricated sensor based on Mach-Zender interferometry was described.. The sensor is based on the coupling of light from a fiber optic into a sputtered ZnO film. A particularly novel aspect of the fabrication was the use of a die saw to form the interface between the end of the fiber optic (set into an anisotropically etched groove) and the ZnO film. Methods to improve the spatial resolution were the subject of a presentation on light addressable potentiometric sensors with SOI and SOS wafers. Silicon on insulator wafers were formed by direct wafer bonding of two silicon wafers, yielding devices with active wafer thicknesses of 10 μm, 100 μm, and 500 μm. The authors also evaluated LAPS devices made using a 0.6 μm silicon layer on a sapphire substrate. Active sensing sites 1 mm by 1 mm in area were defined by selectively doping the silicon layers, and were successfully interrogated by scanning a visible laser.

The second session on this general topic included discussions on a wide range of processes including chemical vapor deposition (CDV) laser ablation, sputtering, evaporation, and etching. One presentation described an elegant approach for defining localized regions of material with a CVD deposition process for SnO_2_, ZnO, TiO_2_, and Pt. Utilizing arrays of micro-machined hotplates, measuring just 100 μm×100 μm, thermal activation of the CVD process provides local film deposition. This is an attractive alternative to lithography for these materials which are difficult to etch. The motivation for this work is to develop fabrication techniques for low cost, multi-component gas sensor arrays that will have low power consumption. A suitable precursor is required for the material to be deposited on the hotplate. The hotplate is instrumented with sensors for temperature and conductivity in addition to the heater film. By momentarily lowering the temperature of the film, the conductivity was monitored as a function of time providing in-process monitoring. The low thermal mass of the hotplates results in a fast time constant of about 1 ms. With a suitable choice of process conditions, the growth rate and film morphology can be controlled. In another presentation, evaporation of nickel onto silicon substrates in an ozone ambient was reported. A linear variation in film stoichiometry was achieved between NiO and NiO_2_ with ozone partial pressure. The films were extensively characterized by TEM, ellipsometry, and Auger spectroscopy. The films responded to various gases with shifts in the work function, measurements were made at 130 °C. The use of laser ablation CVD for the deposit of stoichiometric films was also reported utilizing a KrF eximer laser (248 nm). Al_2_O_3_ was ablated onto oxidized silicon substrates at growth rates between 1 nm/s and 5 nm/s. A 50 nm thick alumina layer deposited at 800 °C was used to form an Electrolyte-Insulator-Semiconductor (EIS) structure and a pH sensor. Capacitance/voltage measurements of the flat-band voltage shift with pH demonstrated a slope close to Nernstian at 55±1 mV per decade over a pH range of 3 to 10. Films were very stable for 600 d with a drift of 0.35 mV/d at pH 7.0. A Japanese group reported on advances in thick film fabrication for SnO_2_ sensors. These devices had one third the power consumption of existing devices at only 280 mW. Various catalytic metals were added to the SnO_2_ to produce sensitivities to various gaseous species including: methane, hydrogen, ethanol to name only a few.

Reduced power consumption and smaller sensor packages have allowed the introduction of new compact portable products. Etching of silicon is a key process in microfabrication and photo-assisted etching that allows greater flexibility and opportunity for control through p-type and n-type doped regions was discussed by an MIT group. High aspect ratio structures have been fabricated. These processes may also be extended to other materials including SiC.

The third and last session on sensor fabrication began with a presentation on the relationship between nonlinear resistance changes in ultra-thin (17 Å to 28 Å) platinum films and their biological responses. A layer of anti-staphylococcal enterotoxin antibody was covalently immobilized onto the electrode surface. The impedance change at 100 Hz upon antigen binding demonstrated direct transduction of the antibody binding with this model system. The films with greater non-linear dc resistance behavior, suggestive of electron tunneling, showed the highest biological response. Another biologically related presentation dealt with the detection of H_2_O_2_ electrochemically. The presentation described the fabrication and characterization of platinum and rhodium working electrodes for use in a novel transcutaneous glucose sensor. Inks evaluated included high- and low-temperature platinum inks, a platinum-carbon ink, and a rhodium-carbon ink. The final presentation discussed a approach for preparing arrays of gold electrodes, each functionalized with a different self-assembled monolayer (SAM). The process exploits reductive desorption to selectively remove a SAM film from an addressed electrode, after which a second SAM film can be deposited. Proof of concept was demonstrated by forming a patterned region of alkanethiol in a field region of a thiol-terminated polyethylene glycol. Anti-bovine serum albumin was selectively adsorbed to the alkanethiol patterns.

### 3.17 Signal Processing Session (reported by M. Koudelka-Hep, Switzerland)

The session on signal processing concentrated on utilizing advanced signal processing techniques and novel interrogation methods for improving sensor performance. During the session, several different types of sensors were discussed and their various shortcomings such as long-term drift, irreproducibility, as well as problems stemming from their lack of characterization and use under non-ideal conditions were considered. It was shown how, through the choice of an adequate signal processing technique, these problems might be overcome and an improved discrimination in model systems obtained. The importance of the sensor characterization and of the influence of different parameters on the optimization of the processing were stressed. Furthermore, the possibility of devising novel techniques for interrogation of sensors was presented for the case of conducting polymer-based sensors. This particular approach consisted in using ac rather then dc measurements. It constitutes another way to improve the sensor array performance, through lowering the detection limits and by increasing the information content.

Summarizing the signal processing session, the importance of thorough sensor characterization in order to be able to exploit the immense possibilities offered by sophisticated signal processing techniques was clearly demonstrated. Together with the use of well defined model systems and of new sensor interrogating techniques, better discrimination and long-term performance of different sensor types should be achieved.

### 3.18 Optical Devices Session (reported by R. Seitz, U.S.A.)

The highlight of this session was the description of a new microfabricated device for measuring surface plasmon resonance by a research team from Texas Instruments Corporation and the University of Washington. This device will only cost a few dollars if it is made in large quantities. In another presentation, a sensitive system for measuring refractive index changes at the surface of an electrode by surface plasmon resonance was also described. The session included descriptions of new indicators for optical detection including separate presentations about indicators for ammonia sensing and heavy metal ion sensing as well as new long wavelength indicators. One presentation discussed the challenges of developing micron scale fiber optic sensors for detecting metal ions, oxygen, and glucose.

One of the more interesting presentations dealt with a portable fiber optic biosensor developed by the Naval Research Laboratory. The discussion included both the arrangements for bringing samples into the reagent clad fiber and how high sensitivity was obtainable. A new type of micromachined optical fiber for use in domestic gas sensors based on absorption of infrared light was also discussed. It is designed to improve the long-term stability of this type of gas sensor. A vibrational spectroscopic technique for detection of phosphonates was also described. There were two presentations on methods involving chemiluminescence. One paper by a Japanese group described a study of catalytic luminescence of gas phase molecules on alumina while the other described a highly sensitive method for detecting hydrazide based on ruthenium chelate chemiluminescence.

## 4. Proceedings

The proceedings of this, The 6th International Meeting on Chemical Sensors, will be published as a special issue in the journal *Sensors and Actuators, Part B*. It is hoped that it will be available for distribution in early 1997.

## 5. Future Meeting

The steering committee of the 6th International Meeting on Chemical Sensors voted unanimously to hold the next meeting in July 1998. The site chosen for the meeting is Beijing, Peoples Republic of China. The new organizing chairperson is Professor Zhi-Gang Zhou, Tsinghua University, Department of Materials Science and Engineering, Beijing, China. Howard H. Weetall, NIST was elected Chairperson of the Steering Committee.

